# Impaired Thiamine Metabolism in Amyotrophic Lateral Sclerosis and Its Potential Treatment With Benfotiamine: A Case Report and a Review of the Literature

**DOI:** 10.7759/cureus.40511

**Published:** 2023-06-16

**Authors:** Richard H Mann

**Affiliations:** 1 Podiatry, Realm Labs, LLC, Boca Raton, USA

**Keywords:** motor neuron disease, thiamin, benfotiamine, vitamin b1, vitamin b1 deficiency, neurodegenerative disease, neurodegeneration, thiamine deficiency, thiamine, amyotrophic lateral sclerosis

## Abstract

Homogenates of brain tissue from the frontal cortex at autopsy in patients with amyotrophic lateral sclerosis (ALS) showed dramatically reduced levels of the enzyme thiamine pyrophosphatase (TPPase), the enzyme responsible for the conversion of thiamine pyrophosphate (TPP) to thiamine monophosphate (TMP). Additionally, free thiamine (vitamin B1) and TMP levels have been shown to be significantly reduced in the plasma and cerebral spinal fluid (CSF) of patients with ALS. These findings suggest that there is impaired thiamine metabolism in patients with ALS. Impaired thiamine metabolism decreases adenosine triphosphate (ATP) production and is a well-established cause of neurodegeneration. Decreased levels of TPPase, resulting in decreased levels of TMP in the cells of the frontal cortex, might account for the focal neurodegenerative changes observed in motor neurons in ALS. Benfotiamine, a safe, lipid-soluble, highly absorbable thiamine analogue, significantly raises free thiamine, TMP, and TPP levels in the blood. A case in which benfotiamine may have positively impacted the symptoms of a patient with ALS is presented. The use of benfotiamine in patients with ALS appears to be a promising therapeutic option. Considering the severity and the lack of satisfactory treatment options associated with this disease, more research on the effects of benfotiamine on the course of ALS is urgently needed.

## Introduction

Adenosine triphosphate (ATP) is essential for the intracellular transfer of energy needed in metabolic processes responsible for the healthy functioning of neurons and all other cell types. The vast majority of cellular ATP is believed to be produced in the mitochondria through the process of oxidative phosphorylation. Oxidative phosphorylation requires the action of Krebs cycle enzymes. Diminished activities of multiple enzymes of the Krebs cycle have been noted following thiamine deficiency [1]. Impairment of normal thiamine metabolism reduces ATP levels and is associated with neurodegeneration [2,3].

Several neurodegenerative diseases have been shown to be associated with impaired thiamine metabolism. Thiamine deficiency results in Wernicke encephalopathy (WE), whose manifestations include cognitive impairment and confabulation [4]. Thiamine supplementation is the cornerstone of the treatment of WE.

Bettendorff et al. [5] found that thiamine pyrophosphate (TPP) levels at autopsy were reduced significantly in the frontal, temporal, parietal, and occipital cortex of six patients with frontotemporal dementia (FTD), suggesting that impaired thiamine metabolism may be a factor in the pathobiology of FTD.

Costantini et al. [6] reported clinical improvement in patients with Parkinson’s disease after parenteral therapy with high doses of thiamine. They inferred that a focal, severe thiamine deficiency due to a dysfunction of thiamine metabolism could cause selective neuronal damage in the centres that are typically affected by Parkinson’s disease. They also reported that high-dose thiamine therapy improved the symptoms of Friedreich’s ataxia in two patients [7] and led to an improvement of motor symptoms in a patient with spinocerebellar ataxia type 2 [8].

Impaired thiamine metabolism has been associated with Alzheimer’s disease (AD). Rao et al. [9] found that the activities of the enzyme TPPase are reduced by 62% in the temporal cortex and 28% in the frontal cortex in patients with AD. Gibson et al. [10] found that the activities of thiamine-dependent enzymes appear to be deficient in patients with AD. They found the activity of the thiamine-dependent Krebs cycle enzyme 2-ketoglutarate dehydrogenase complex to be dramatically reduced in brain homogenates of deceased patients with AD. Pan et al. [11] found that patients with AD had significantly reduced blood TPP levels as compared to control subjects. Researchers from three separate groups reported improvement in cognitive function in patients with AD following therapy with benfotiamine, a safe, lipid-soluble, highly absorbable thiamine analogue that significantly raises blood thiamine levels [12-15]. The improvement observed in the symptoms of these patients after the administration of benfotiamine suggests that benfotiamine may improve thiamine metabolism in the brains of patients with AD.

Diabetic polyneuropathy and alcoholic polyneuropathy, both neurodegenerative diseases of the peripheral nervous system, are associated with thiamine deficiency and have been shown to improve with the use of benfotiamine [16,17].

Amyotrophic lateral sclerosis (ALS) has been associated with impaired thiamine metabolism [18-20]. As illustrated in the following case presentation, the use of benfotiamine in the treatment of patients with ALS represents a potentially promising therapeutic option.

## Case presentation

A 69-year-old white male with a diagnosis of slow-progressing ALS of one year’s duration claimed to be in less pain and to have significantly less fatigue within four days of initiating a course of 300 mg of benfotiamine and 1 mg of methylcobalamin (BAM) twice a day by mouth. He also claimed a sense of improved cognition. His sense of greater muscular strength and improved cognition remained unchanged for approximately eight weeks of taking BAM daily. At the end of the eight-week course, he discontinued taking BAM and claimed that within four days his symptoms had regressed to where they had been prior to initiating BAM. The patient then resumed taking BAM and claimed that within two days he experienced a resumption of his sense of greater muscular strength and improved cognition.

## Discussion

**Figure 1:**
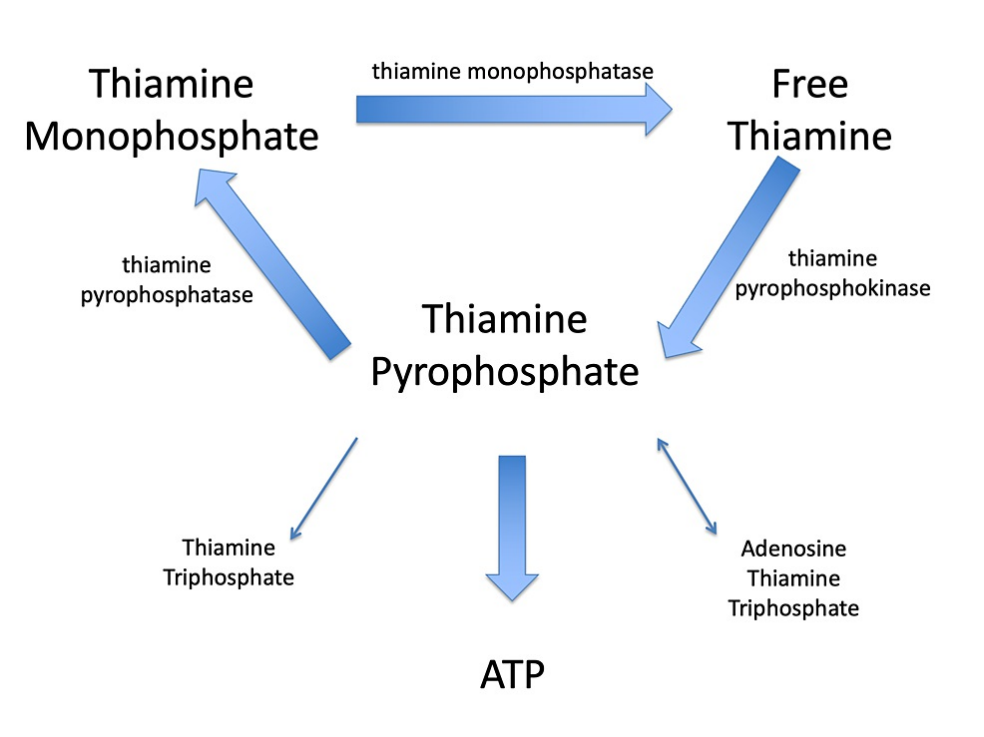
Thiamine metabolism. In healthy thiamine metabolism, free thiamine is converted to thiamine pyrophosphate (TPP) through the actions of thiamine pyrophosphokinase (TPK). TPP is converted to thiamine monophosphate (TMP) via the actions of thiamine pyrophosphatase (TPPase). TMP is converted to free thiamine via the actions of thiamine monophosphatase (TMPase). TPP is essential for adenosine triphosphate (ATP) production and normal cellular functioning. It acts as a coenzyme for enzymes of the Krebs cycle and other metabolic pathways. TPP may also be converted to thiamine triphosphate and adenosine thiamine triphosphate. The biological significance of these thiamine triphosphate forms does not appear to be related to ATP production and their functions are not well understood.

Thiamine, also known as free thiamine, is essential for all animal life and the proper functioning of all cell types. It is not created by the body and must be obtained from food. After thiamine is absorbed from the gut, it enters the bloodstream, where it is subsequently transported into neurons and all other cell types. Under normal circumstances, during its intracellular metabolism, thiamine is converted to its biologically active form, TPP, through the actions of the enzyme thiamine pyrophosphokinase (TPK). TPP is then converted to thiamine monophosphate (TMP) through the action of the enzyme thiamine pyrophosphatase (TPPase). TMP is then converted to free thiamine through the action of the enzyme thiamine monophosphatase (TMPase). As such, thiamine metabolism is, in part, cyclical in nature [21,22]. The cycle flows from free thiamine to TPP to TMP, and to free thiamine again (Figure 1).

Impaired thiamine metabolism in ALS 

Homogenates from the frontal cortex of deceased ALS patients showed significantly lower levels of TPPase than controls in research by Laforenza et al. [18]. Poloni et al. [19] showed that patients with ALS have decreased levels of thiamine and TMP in the CSF, suggesting decreased TMP levels in the central nervous system cells. They also found decreased levels of thiamine and TMP in the plasma of patients with ALS. They describe ALS as a dying-back process, with the largest and longest nerve fibres often being affected earliest in the disease. This is a similar pattern to what is also observed in the polyneuropathy of thiamine deficiency as well - the longest fibres are affected earliest in the disease [23].

Probert et al. [24] showed that in a transgenic mouse model of ALS, the thiamine derivative O,S-dibenzoyl thiamine improved physiological outcomes, motor function, and muscle atrophy compared to vehicle. Jesse et al. [20] reported morphological signs of acute WE in the autopsy of the brains of two patients with ALS. They further found thiamine deficiency in 28% of the 122 patients with ALS. Fourteen of these 122 patients had mild thiamine deficiency, and 20 of these patients had severe thiamine deficiency. Jhala and Hazell [25] reported thiamine deficiency to be a well-established cause of neurodegeneration and found emerging evidence suggesting that thiamine deficiency produces alterations in brain function and structural damage that closely model several maladies in which neurodegeneration is a characteristic feature, including ALS. In their analysis of the available scientific literature, Goncharova et al. [26] found that thiamine was likely to reduce the risk of ALS and had a protective effect.

Decreased availability of TPPase within motor neurons in ALS would impair thiamine metabolism, causing reduced efficiency in Krebs cycle enzymes, decreased ATP production, and neurodegeneration. A possible cause of decreased TPPase levels is the well-documented phenomenon of fragmentation and atrophy of the Golgi apparatus observed in the motor neurons of patients with ALS [27]. TPPase is highly concentrated in the Golgi apparatus [28]. A disruption in the integrity of the Golgi apparatus would likely cause decreased availability of TPPase and subsequent impaired thiamine metabolism. Whether from Golgi fragmentation and atrophy or some other phenomenon, the evidence is consistent with the hypothesis that decreased TPPase availability in the motor neurons of patients with ALS may decrease the ability of these cells to produce ATP and cause neurodegeneration.

Benfotiamine and ALS

Analysis of the therapeutic effect of methylcobalamin on the symptoms of ALS would suggest that, although methylcobalamin at high doses may have some beneficial effect, the perceived rapid improvement in symptoms experienced by the patient described in this case presentation is from the actions of benfotiamine and not methylcobalamin [29].

**Figure 2:**
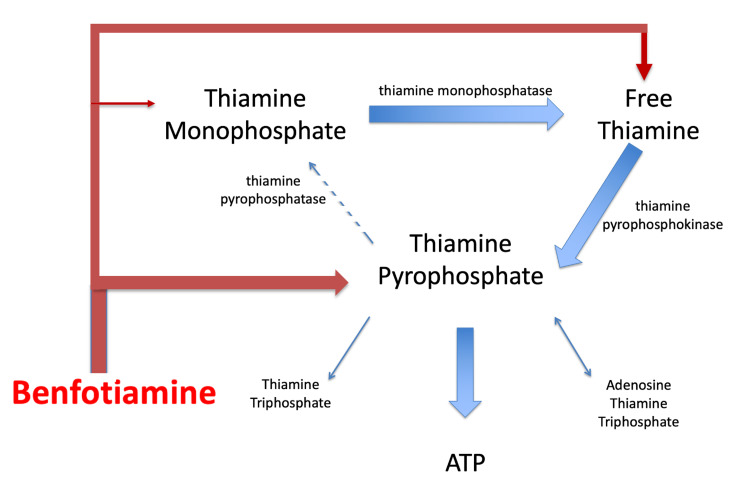
The potential effect of benfotiamine on impaired thiamine metabolism in ALS. Benfotiamine increases levels of free thiamine, TMP, and TPP thereby improving thiamine metabolism with the likely effect of countering the impaired thiamine metabolism that occurs in ALS due to decreased activity of thiamin pyrophosphatase.

Benfotiamine has been shown to raise free thiamine, TMP, and TPP levels in the blood to a much greater degree than direct thiamine administration [14,15]. Increased availability of free thiamine and thiamine phosphates could potentially counter the effects of the impaired thiamine metabolism associated with ALS (Figure 2).

This would have the effect of restoring normal thiamine metabolism and increasing ATP production. Increased ATP levels in motor neurons could potentially counteract the neurodegenerative processes observed in ALS, possibly improving neuronal function and inhibiting disease progression.

## Conclusions

Impaired thiamine metabolism appears to be involved in several neurodegenerative diseases. The evidence suggests that this may be the case in ALS. In ALS, the neurons of the frontal cortex have been shown to have decreased levels of the enzyme TPPase. Decreased TPPase activity is consistent with decreased TMP levels in the CSF of patients with ALS. The disruption in healthy thiamine metabolism would likely cause decreased efficiency of Krebs cycle enzymes required for oxidative phosphorylation, leading to decreased ATP production. This, in turn, may potentially cause the focal neurodegeneration of these motor neurons. The mechanism by which TPPase levels are reduced in these cells may be due to the well-documented finding in ALS of golgi fragmentation and atrophy, or the reduction may be due to some other yet-to-be-determined process. In any event, the pathobiological effects of impaired thiamine metabolism may be countered by benfotiamine. Although the improvement noted by the patient in this case presentation may be due to a placebo effect, the rapid improvement experienced by the patient after initiating BAM therapy, the regression of symptoms experienced after he discontinued BAM, and his perceived rapid resumption of improvement after restarting BAM further support this hypothesis.

Considering the well-established association between impaired thiamine metabolism and neurodegeneration, the evidence that impaired thiamine metabolism is associated with ALS, and the paucity of satisfactory options currently available for the treatment of ALS, further research on the potential therapeutic benefits of benfotiamine is urgently needed.
